# The association between student body mass index and tests of flexibility assessed by the FITNESSGRAM®: New York City public school students, 2017–18

**DOI:** 10.1371/journal.pone.0262083

**Published:** 2021-12-31

**Authors:** Hannah R. Thompson, Andjelka Pavlovic, Emily D’Agostino, Melanie D. Napier, Kevin Konty, Sophia E. Day

**Affiliations:** 1 Community Health Sciences, School of Public Health, University of California Berkeley, Berkeley, California, United States of America; 2 Division of Youth Education, The Cooper Institute®, Dallas, Texas, United States of America; 3 Department of Population Health Sciences, Department of Family Medicine & Community Health, Duke University School of Medicine, Durham, North Carolina, United States of America; 4 Department of Epidemiology, University of North Carolina, Chapel Hill, North Carolina, United States of America; 5 Office of School Health, New York City Department of Health and Mental Hygiene, New York City, New York, United States of America; Weill Cornell Medical College in Qatar, QATAR

## Abstract

FITNESSGRAM® is the most widely used criterion-referenced tool to assess/report on student health-related fitness across the US. Potential weight-related biases with the two most common tests of musculoskeletal fitness–the trunk extension and Back-Saver Sit-and-Reach (sit-and-reach)—have been hypothesized, though have not been studied. To determine the association between musculoskeletal fitness test performance and weight status, we use data from 571,133 New York City public school 4^th^-12^th^ grade students (85% non-White; 75% qualified for free or reduced-price meals) with valid/complete 2017–18 FITNESSGRAM® data. Adjusted logistic mixed effects models with a random effect for school examined the association between weight status and whether a student was in the Healthy Fitness Zone (HFZ; met sex- and age-specific criterion-referenced standards) for the trunk extension and sit-and-reach. Compared to students with normal weight, the odds of being in the HFZ for trunk extension were lower for students with underweight (OR = 0.77; 95% CI: 0.741, 0.795) and higher for students with overweight (OR = 1.10; 95% CI: 1.081, 1.122) and obesity (OR = 1.11; 95% CI: 1.090, 1.13). The odds of being in the HFZ for sit-and-reach were lower for students with underweight OR = 0.85; 95% CI: 0.826, 0.878), overweight (OR = 0.83; 95% CI: 0.819, 0.844) and obesity (OR = 0.65; 95% CI: 0.641, 0.661). Students with overweight and obesity perform better on the trunk extension, yet worse on the sit-and-reach, compared to students with normal weight. Teachers, administrators, and researchers should be aware of the relationship of BMI with student performance in these assessments.

## Introduction

Adequate physical fitness in childhood and youth is not only critical for optimal present-day health, but is an important predictor of future health outcomes [1, 2]. The FITNESSGRAM®, a multi-component physical fitness assessment, is the most widely-used tool to assess and report on the fitness and physical activity of primary and secondary students across the United States [3]. The FITNESSGRAM’s® tests of aerobic capacity, musculoskeletal fitness (including muscle strength, muscular endurance, and flexibility), and body composition are used to measure health-related physical fitness and weight status. The results of these assessments are utilized to promote lifelong fitness and physical activity in more than 22 million school-aged US children and youth across all 50 states [3].

While numerous rigorous, large-scale studies have been conducted to assess the validity and reliability, as well as determine the criterion-referenced standards of the FITNESSGRAM® tests for body composition and aerobic capacity [4–6]—the two components of youth fitness demonstrating the strongest links between physical, mental, and academic health outcomes [7–9]—far fewer studies have determined the validity and reliability of the musculoskeletal fitness tests in school-aged youth [10–13]. Specifically, there’s a paucity of evidence on the commonly used trunk extension and Back-Saver Sit-and-Reach (sit-and-reach) tests.

Establishing a link between flexibility and health outcomes has been difficult, for several reasons. While other fitness components are more systemic in nature, flexibility is highly specific to each joint, such that flexibility in one joint says nothing about flexibility in another [14]. Flexibility may also not be linearly related to health outcomes [14]. However, the FITNESSGRAM® includes these tests due to the importance for youth to learn about flexibility and its usefulness for lifelong health [3].

Flexibility, the ability to move body components (i.e. joints, tendons, muscles, tissues) through a range of motion, is considered an important dimension of youth fitness [5, 15, 16], primarily because it is hypothesized to be associated with prevention of/relief from lower-back pain, avoidance of musculoskeletal injury, and improved posture [14]. However, there is little empirical evidence linking flexibility and health in youth [3, 14]. Reliability and validity studies of the trunk extension and sit-and-reach tests have been conducted in school-aged populations, with mixed results [10, 11, 17–19]. For example, the sit-and-reach was found to be a valid test for evaluation of pelvic tilt and lumbar flexion (though invalid for measuring hamstring flexibility) in 6–18 year-olds [11], while the trunk extension test has been deemed invalid for identifying students with lower back pain [10].

For the trunk extension test, the student is asked to lay on his/her stomach and lift his/her trunk as in a controlled manner; his/her score is recorded as the number of inches he/she is able to lift off the ground (not to exceed 12 inches) [3]. While this is intended to measure both minimal trunk extensor strength and lumbar flexibility, as well as to be used as an indicator of lower back pain [3, 20], anecdotal observations from physical education teachers and school- and district-level administrators tasked with collecting and interpreting FITNESSGRAM® data, as well as one small study in Texas [21], suggests this test may be biased towards students with higher body composition.

For the sit-and-reach test, which is used to measure lower body (lumbar spine and hamstring) flexibility [3], students sit on the floor with one leg extended straight in front of them with their foot against a sit-and-reach box and the opposite leg bent with the foot flat on the floor. The student reaches forward for a measurement of that side, and then repeats on the other side. The measurement is recorded with a maximum outcome of 12 inches. As with the trunk extension test, concerns about the impact of student adiposity on test performance have also arisen [3]. The extent to which student weight status is associated with a student’s sit-and-reach performance similarly remains unknown.

Several states (California, Delaware, Georgia, Illinois, Kansas, South Carolina, Texas, and Vermont) and many large school districts mandate coordinated youth fitness testing by the FITNESSGRAM® [22]. The New York City Department of Education (NYCDOE), the largest and among the most racially/ethnically and socio-economically diverse school districts in the nation [23], uses the FITNESSGRAM® annually to assess fitness among its approximately 1.1 million students. The New York City Department of Health and Mental Hygiene (NYCDOHMH), which manages, analyzes, and reports on the NYCDOE’s FITNESSGRAM® data [24], has never reported on student trunk extension and sit-and-reach data, due to hypothesized potential weight-related biases with these tests. However, empirical evidence on the association between musculoskeletal flexibility test performance and student weight status is lacking. If students are being classified as physically fit on the trunk extension test based on their body composition (and not on trunk strength), this may harm, rather than help, students’ understanding of fitness.

The purpose of this study was to examine the association between individual students’ weight status, as determined by their body mass index (BMI) and 1) trunk extension scores and 2) sit-and-reach scores in a large and highly diverse sample of 4^th^-12^th^ grade students. Results from this study could help inform the future use and interpretation of FITNESSGRAM® flexibility test data from primary and secondary school students.

## Methods

### Data source and study population

Data for this study were drawn from the NYC FITNESSGRAM dataset [24, 25] jointly managed by NYCDOE and NYCDOHMH for the 2017–18 school year (the most recent year available). This dataset included annual fitness assessment data collected by NYCDOE for 1,079,542 NYC public school students (grades kindergarten -12) [26]. Inclusion criteria for this study were: 1) enrollment in the 4^th^–12^th^ grades (grades in which both the flexibility and body composition assessments are conducted, which excluded 332,199 students); and 2) enrollment in traditional education districts (excluding 3 districts which educated special education or adult students and are not required to administer the FITNESSGRAM®, which excluded an additional 94,526 students). A total of 652,817 students were eligible for inclusion, ranging from 7 to 19 years of age. Both the NYCDOHMH Institutional Review Board and the UC Berkeley Committee for the Protection of Human Subjects deemed this non-human subject research.

### FITNESSGRAM® measures

The FITNESSGRAM® is administered from September–May each school year by physical education teachers who receive formal training on conducting the test, including manuals, video-based training, and site visits, as well as required equipment [26]. NYCDOE schools are mandated to have ≥85% of eligible students complete the FITNESSGRAM® assessment each year.

#### Flexibility measurements

In the NYCDOE, assessment of student flexibility begins in grade 4, continues through grade 12, and follows recommended FITNESSGRAM® protocol [27]. For the trunk extension test, a student laid down on a mat, in the prone (facedown) position, with toes pointed and hands placed under the thighs. A coin or other marker was placed on the floor, in line with the student’s eyes. While maintaining focus on the coin, the student lifted the upper body off the floor and held the position until the teacher placed a ruler on the floor in front of the student and measured the distance from the floor to the student’s chin. A second trial was permitted, with the highest of the 2 scores being recorded. Students’ trunk extension scores were recorded in number of inches (with a maximum of 12 inches). A student was considered to be in the Healthy Fitness Zone (HFZ) for trunk extension if their score met or exceeded the determined age- and sex-specific criteria established by The Cooper Institute [3].

For the sit-and-reach test, students were assessed using a sit-and-reach box (a sturdy box approximately 12 inches high with a ruler extending off the top, parallel to the floor). With one leg bent with foot flat on the floor and the other leg extended with foot flat against the face of the box, the student was instructed to bend forward with their arms, palms down, one on top of the other, extended over the ruler. The student reached directly forward (maintaining a straight back and keeping the head up) with both hands along the ruler 4 times and held the position of the 4^th^ reach for at least 1 second; a measurement in inches was recorded (maximum 12 inches). After one side was measured, the procedure was repeated on the second side. A student was considered to be in the HFZ for sit-and-reach if his/her scores met or exceeded the determined age- and sex-specific criteria established by the Cooper Institute for both sides (left and right) [3].

#### Body mass index measurements

Body composition was measured by body mass index (BMI) and most commonly assessed using a combined scale and stadiometer (Health-O-Meter 500 KL). Per the Cooper Institute protocol [3], height was measured to the nearest 0.1 inch twice, and if the two measures were off by more than 0.5 inch, a third height was recorded. Weight was measured to the nearest integer pound. Student birth date was used to calculate students’ exact age in months and together with student sex, height, and weight, was converted to age- and sex-specific BMI percentile using the Center for Disease Control and Prevention (CDC) clinical growth charts [28]. As defined by the CDC [29], a child’s weight status was classified as: underweight (BMI-for-age < 5^th^ percentile); healthy weight (BMI-for-age ≥ 5^th^ and < 85th percentile); overweight (BMI-for-age ≥ 85^th^ and < 95^th^ percentile); or obese (BMI-for-age ≥ 95^th^ percentile).

### Covariates

Available known correlates of physical activity, gross motor competence, and weight status in youth were included as covariates [30–33], including: student-level sex; race/ethnicity; age; place of birth; home language; and eligibility for free or reduced-price meals (FRPM) through the National School Lunch Program, used as a proxy for socio-economic status (provided for all students via parental report); student-level grade and Individualized Education Program (IEP) participation (academic disability) status (provided via school records); and school grade levels offered (elementary (grades 4^th^-5^th^), middle (6^th^-8^th^) and high (9^th^-12^th^); also provided via school records).

### Statistical analysis

In order to determine the association between individual students’ weight status and the odds of that student being in the HFZ for the 1) trunk extension test and 2) sit-and-reach test, we used logistic mixed effects models accounting for clustering by school. Models adjusted for student-level race/ethnicity, place of birth, home language, FRPM eligibility, grade, and academic disability status. Models were also run stratified by key student-level demographic characteristics that could modify the association between body composition and flexibility [30]: sex, grade category (elementary (grades 4–5), middle (grades 6–8), high (grades 9–12)), race/ethnicity, and FRPM eligibility status. In a sensitivity analysis, models were run with students being classified as in the HFZ for the sit-and-reach if they met HFZ standards for at least on side (either left, right, or both sides). Analyses were conducted in Stata/MP 16.1 (StataCorp, College Station, Texas).

## Results

The final analytic sample included 571,133 students (87.5% of eligible sample) who had valid/complete BMI and either trunk extension or sit-and-reach data; 570,003 students (87.3%) had both trunk extension and BMI data; 569,991 (87.3%) had both sit-and-reach and BMI data. Compared to students with complete data, students with missing/non-valid data were more likely to be male (53% male with missing/non-valid vs. 51% male with complete data); less likely to be Asian/Pacific Islander (12% vs. 19%) or white (12% vs. 16%); more likely to be Non-Hispanic Black (30% vs. 23%) or Hispanic (44% vs. 40%); more likely to qualify for FRPM (77% vs. 75%); more likely to have an IEP (25% vs. 18%); more likely to be foreign-born (25% vs. 20%); and less likely to speak English at home (43% vs. 46%; p-values for all tests <0.001).

Student sample demographic characteristics are described in Table 1. This is a highly racially/ethnically (19% Asian/Pacific Islander; 23% non-Hispanic Black; and 40% Hispanic) and socioeconomically (75% of students qualified for FRPM) diverse sample. Mean student age was 13.6 (SD ± 2.7) years. Nearly a quarter (24%) of students were in elementary grades (4–5); 33% were in middle school grades (6–8); and 43% were in high school grades (9–12), with an average of 63,459 students per grade.

**Table 1:** Demographic characteristics of New York City public school 4^th^-12^th^ grade students with FITNESSGRAM® data (N = 571,133), 2017–18.

	n	%
Sex		
Male	280,319	49.1
Female	290,814	50.9
Grade levels		
Elementary grades (4^th^-5^th^)	136,665	23.9
Middle school grades (6^th^-8^th^)	189,391	33.2
High school grades (9^th^-12^th^)	245,077	42.9
Race/Ethnicity		
Hispanic	108,526	19.0
Non-Hispanic black	132,707	23.2
Asian and/or Pacific Islander	229,411	40.2
Non-Hispanic white	89,930	15.8
Other/missing	10,559	1.9
Primary language spoken at home		
English	308,181	54.0
Spanish	141,764	24.8
Other language	121,169	21.2
Place of birth		
U.S.A	456,882	80.0
Outside the U.S.A.	114,251	20.0
Student socioeconomic status		
Does not qualify for free or reduced-price meals	140,709	24.6
Qualifies for free or reduced-price meals	430,424	75.4
Academic need		
Students without individualized educational plans	467,734	81.9
Students with individualized educational plans	103,399	18.1
Body mass index^A^		
Underweight (<5^th^ percentile)	20,931	3.7
Normal weight (5th—<85^th^ percentile)	336,026	58.8
Overweight (85^th^—<95^th^ percentile)	103,975	18.2
Obese (< = 95^th^ percentile)	110,201	19.3
Trunk Extension		
In the Healthy Fitness Zone^B^	430,623	75.5
Back-saver sit-and-reach		
In the Healthy Fitness Zone (left and right sides)	325,507	57.1
In the Healthy Fitness Zone (for at least one side)	367,282	64.4

^A^Body mass index percentiles specific to age and sex calculated based on U.S. Center for Disease Control growth charts

^B^ The FITNESSGRAM® uses Healthy Fitness Zones—criterion-referenced standards which represent minimum levels of fitness for age and sex that offer protection against the diseases that result from sedentary living–to evaluate fitness performance of students.

Four percent of students were classified as having underweight; 59% as having normal weight; 18% as having overweight; and 19% as having obesity. Students were more likely to be in the HFZ for the trunk extension (76%), as compared to for the sit-and-reach (57% for both sides; 64% for at least one side). Sixty-three percent of students were in the HFZ for both the trunk extension and for the sit-and-reach for both sides.

The odds of being in the HFZ for trunk extension were lower (OR = 0.77; 95% CI: 0.741, 0.795) for students with underweight and higher for students with overweight (OR = 1.10; 95% CI: 1.081, 1.122) and with obesity (OR = 1.11; 95% CI: 1.090, 1.13) compared to students with normal weight (Table 2). This association appeared to hold true in stratified models among all subgroups with two exceptions: there was no statistically significant association between weight status and being in the HFZ for trunk extension for high school students with obesity compared to high school students with normal weight (OR: 1.0; 95% CI: 0.973, 1.033) nor for non-Hispanic Black students with overweight compared to non-Hispanic black students with normal weight (OR = 1.02; 95% CI: 0.982, 1.055).

**Table 2:** Adjusted associations^A^ between student weight status^B^ and odds of being in the healthy fitness zone^C^ for the FITNESSGRAM® tests for flexibility among New York City 4^th^-12^th^ grade public school students, 2017–18, by student-level demographic characteristics.

	Trunk Extension (n = 569,984 students)						Back-Saver Sit-and-Reach (n = 569,972 students)					
	Underweight		Overweight		Obese		Underweight		Overweight		Obese	
	# of students	OR ± SE (95% CI)	# of students	OR ± SE (95% CI)	# of students	OR ± SE (95% CI)	# of students	OR ± SE (95% CI)	# of students	OR ± SE (95% CI)	# of students	OR ± SE (95% CI)
All students	20,896	0.77 ± 0.01 (0.741, 0.795)	103,749	1.10 ± 0.01 (1.081, 1.122)	109,946	1.11 ± 0.01 (1.090, 1.13)	20,888	0.85 ± 0.01 (0.826, 0.878)	103,763	0.83 ± 0.01 (0.819, 0.844)	109,941	0.65 ± 0.01 (0.641, 0.661)
**Stratified models**												
Females	8,869	0.81 ± 0.02 (0.761, 0.853)	52,415	1.10 ± 0.02 (1.071, 1.131)	47,492	1.09 ± 0.02 (1.059, 1.121)	8,867	0.79 ± 0.02 (0.757, 0.834)	52,437	0.82 ± 0.01 (0.807, 0.843)	47,483	0.61 ± 0.01 (0.599, 0.628)
Males	12,027	0.77 ± 0.02 (0.738, 0.809)	51,334	1.11 ± 0.01 (1.086, 1.143)	62,454	1.18 ± 0.01 (1.152, 1.209)	12,021	0.76 ± 0.02 (0.726, 0.791)	51,326	0.85 ± 0.01 (0.830, 0.869)	62,458	0.61 ± 0.01 (0.594, 0.621)
Elementary (Grades 4–5)	5,487	0.76 ± 0.03 (0.701, 0.816)	25,603	1.17 ± 0.02 (1.127, 1.220)	31,493	1.30 ± 0.03 (1.256, 1.355)	5,486	0.85 ± 0.03 (0.796, 0.906)	25,611	0.79 ± 0.01 (0.769, 0.822)	31,462	0.57 ± 0.01 (0.554, 0.590)
Middle School (Grades 6–8)	6,450	0.82 ± 0.03 (0.768, 0.869)	36,799	1.15 ± 0.02 (1.120, 1.191)	39,569	1.23 ± 0.02 (1.197, 1.272)	6,449	0.78 ± 0.22 (0.741, 0.828)	36,809	0.86 ± 0.01 (0.839, 0.884)	39,597	0.66 ± 0.01 (0.647, 0.682)
High School (Grades 9–12)	8,959	0.75 ± 0.02 (0.707, 0.789)	41,347	1.07 ± 0.02 (1.038, 1.101)	38,884	1.00 ± 0.02 (0.973, 1.033)	8,953	0.74 ± 0.02 (0.701, 0.773)	41,343	0.83 ± 0.01 (0.813, 0.854)	38,882	0.60 ± 0.01 (0.581, 0.612)
Asian/Pacific Islander	6,862	0.78 ± 0.02 (0.733, 0.830)	17,872	1.22 ± 0.03 (1.167, 1.273)	12,345	1.41 ± 0.04 (1.343, 1.488)	6,859	0.80 ± 0.02 (0.759, 0.848)	17,873	0.77 ± 0.01 (0.744, 0.802)	12,346	0.60 ± 0.01 (0.574, 0.627)
Non-Hispanic black	3,942	0.84 ± 0.03 (0.773, 0.908)	23,292	1.06 ± 0.02 (1.020, 1.102)	27,996	1.02 ± 0.02 (0.982, 1.055)	3,943	0.84 ± 0.03 (0.781, 0.902)	23,287	0.85 ± 0.01 (0.826, 0.882)	27,991	0.62 ± 0.01 (0.604, 0.643)
Hispanic	5,549	0.78 ± 0.03 (0.726, 0.831)	46,482	1.09 ± 0.02 (1.057, 1.118)	55,576	1.12 ± 0.02 (1.087, 1.146)	5,544	0.83 ± 0.03 (0.783, 0.883)	46,494	0.85 ± 0.01 (0.829, 0.869)	55,569	0.61 ± 0.01 (0.596, 0.624)
Non-Hispanic white	3,935	0.80 ± 0.04 (0.733, 0.871)	14,406	1.09 ± 0.03 (1.037, 1.149)	12,378	1.27 ± 0.04 (1.201, 1.343)	3,934	0.76 ± 0.03 (0.711, 0.819)	14,412	0.81 ± 0.01 (0.782, 0.848)	12,383	0.64 ± 0.01 (0.615, 0.670)
Not FRPM eligible	6,222	0.80 ± 0.03 (0.743, 0.853)	23,002	1.10 ± 0.02 (1.061, 1.150)	20,922	1.18 ± 0.03 (1.136, 1.236)	6,226	0.78 ± 0.02 (0.739, 0.829)	23,006	0.81 ± 0.01 (0.779, 0.832)	20,928	0.62 ± 0.01 (0.603, 0.646)
FRPM eligible	14,674	0.78 ± 0.02 (0.751, 0.816)	80,747	1.11 ± 0.01 (1.083, 1.129)	89,024	1.13 ± 0.01 (1.106, 1.152)	14,662	0.81 ± 0.02 (0.784, 0.845)	80,757	0.83 ± 0.01 (0.817, 0.847)	89,013	0.61 ± 0.01 (0.597, 0.618)

^A^ Calculated from logistic mixed effects models with a random effect for school, adjusted for student-level: grade, race/ethnicity, free or reduced-price meal eligibility (a proxy for socioeconomic status), academic disability status, primary language spoken at home, and place of birth. The reference category for all models is students with normal weight.

^B^Weight status determined by student body mass index percentiles specific to age and sex calculated based on U.S. Center for Disease Control growth charts and weight status classifications.

^C^ The FITNESSGRAM® uses Healthy Fitness Zones—criterion-referenced standards which represent minimum levels of fitness for age and sex that offer protection against the diseases that result from sedentary living–to evaluate fitness performance of students.

*Abbreviations: OR = Odds Ratio; SE = Standard Error; CI = Confidence Interval; FRPM = Free or reduced-price meal.

The odds of being in the HFZ for sit-and-reach (for both the left and right side) were lower (OR = 0.85; 95% CI: 0.826, 0.878) for students with underweight, with overweight (OR = 0.83; 95% CI: 0.819, 0.844) and with obesity (OR = 0.65; 95% CI: 0.641, 0.661) compared to students with normal weight, with results remaining consistent across stratified models (Table 2). Results from the sensitivity analysis showed similar findings: the odds of being in the HFZ for sit-and-reach (for both sides or just one side) were lower (OR = 0.85; 95% CI: 0.829, 0.873) for students with underweight, with overweight (OR = 0.84; 95% CI: 0.822, 0.848) and with obesity (OR = 0.66; 95% CI: 0.645, 0.666) compared to students with normal weight (S1 Table).

## Discussion

This is the first known study to examine the association between elementary through high school students’ flexibility and weight status, using data from the FITNESSGRAM® trunk extension, sit-and-reach, and body composition tests. Examining data from nearly 570,000 NYCDOE 4^th^-12^th^ grade students (85% non-white and 75% of whom qualify for FRPM), we found statistically significant associations between both trunk extension and sit-and-reach scores and weight status. Whereas students with overweight and obesity did not perform as well on the sit-and-reach test compared to normal weight students, students with overweight and obesity had a greater odds of meeting age- and sex-based performance standards for the trunk extension test.

The odds of being in the HFZ for trunk lift extension were lower (OR = 0.77) for students with underweight, but higher for students with overweight (OR = 1.10) and obesity (OR = 1.11). This held consistent in stratified models among all subgroups except for high school students with obesity compared to high school students with normal weight (OR: 1.0) and for non-Hispanic Black students with overweight compared to non-Hispanic Black students with normal weight (OR = 1.02). It is possible data quality could be driving these null findings, as there has been prior concern about the quality of the NYCDOE’s BMI data among high school students, which is of general lower quality compared to that of Kindergarten– 8th graders. High school NYCDOE students are more likely to be allowed to self-report their BMI, with self-reported measurements demonstrating lower weight and taller height than actual measurements, resulting in biased BMI data with reported BMI lower than actual BMI [25, 34]. Despite this concern, among high school (grade 9–12) students in this sample, 16.9% had overweight and 15.9% had obesity, which closely mirrors what is reported nationally; according to 2019 Youth Risk Behavior Surveillance System data, 16.1% of U.S. high school students had overweight and 15.5% had obesity [35]. Further, if high school students’ are under-reporting their BMI, our current findings are likely conservative; a smaller proportion of high school students with normal weight in our sample would likely bias our findings away from the null. Still, further exploration of this is necessary; results from high school students should be interpreted with caution.

As hypothesized by Ajisafe et al [21], it is possible that students with overweight and obesity have physiologically larger abdomens and backs (the cross-sectional body parts involved in the trunk extension test) and/or need to perform a shorter vertical lift to achieve HFZ status (since they are already elevated off the testing matt due to central adiposity), which puts them at an advantage on the trunk extension test. As a counter theory, it is also possible that children with overweight and obesity have greater rotational trunk inertia and that the resulting increased demand to uphold an upright trunk in the sagittal plane may lead to greater relative trunk extensor strength and flexibility compared to for students with normal weight [21]. Physiological mechanisms for students with underweight having poorer trunk lift performance have not been previously hypothesized. However, underweight has been previously deemed a determinant of health-related fitness in adolescents [36]. Together, these findings suggest further work remains imperative to best understand how to interpret the results of this test.

The odds of being in the HFZ for sit-and-reach were lower for students with underweight (OR = 0.85), overweight (OR = 0.83), and obesity (OR = 0.65) compared to students with normal weight. These findings held consistent across all examined sub-groups of students. The findings for students with overweight and obesity make physiological sense; students with greater adiposity would be expected to have a more difficult time bending forward to perform the sit-and-reach test compared to students with normal weight. However, further study is necessary to better understand why underweight students in this sample demonstrated a lower odds of being in the HFZ for the sit-and-reach compared to normal weight students.

Evidence from smaller studies on elementary students corroborates these findings. Findings from a sample of 415 Kindergarten– 5^th^ grade public school students in Texas [21], similarly demonstrated that higher trunk extension test scores were associated with increased odds of being obese and that higher sit-and-reach scores were associated with a decreased odds of being obese as compared to normal weight (however they did not see an association for either test in students with overweight vs. normal weight). In a study among 3,700 Portuguese students ages 6–10, Pereira et al found that children with overweight were 2.7 times more likely to be in the HFZ for the trunk extension compared to students with normal weight [37]. Neither of these studies presenting findings on students with underweight.

Data from the FITNESSGRAM® tests for flexibility are not often used as singular outcomes or predicters in youth health studies [38, 39]. However, additive scores indicating the number of tests (0–6) for which a student is in the HFZ, or binary scores indicating if a student has met a threshold of being in the HFZ for a certain number of tests (i.e. 5 or 6), have been used as predictors and outcomes in multiple studies examining the relationship between physical activity interventions, academic outcomes, and youth health [40–42]. The findings from this current study suggest that including a students’ HFZ status for the trunk extension test in an additive or threshold score may introduce bias, and thus such analyses should be interpreted with caution.

It is important to note that while FITNESSGRAM® is often used as a surveillance tool, its primary focus is on education and promotion of physical activity and health-related fitness among youth [3]. Despite limited evidence linking flexibility to specific youth health-related outcomes (i.e., reduced low back pain, etc.) [10, 43], the FITNESSGRAM® recognizes flexibility as an important dimension of health-related fitness. The FITNESSGRAM® includes these tests due to the perceived value for youth to learn about flexibility and its importance for lifelong health [3]. Flexibility is also believed to enhance and maintain current physical activity experiences [44]; Chen et al found that health-related fitness component (including trunk extension) performance was associated with 5^th^ grade students’ engagement in both school-day (i.e. physical education and recess) and out of school (i.e. dance and sports) physical activities [39]. However, if students are being classified as in the HFZ for the trunk extension based on their body composition (and not just on trunk strength), this may harm, rather than help, students’ understanding of fitness. The FITNESSGRAM® manual, itself, states, “More research is needed to develop an acceptable trunk extension test [3].” Together these findings indicate more research is indeed needed.

### Limitations

Several study limitations deserve mention. First, although 88% of eligible students had complete FITNESSGRAM® data, there were significant demographic differences between students with and without complete data, which could impact the generalizability of these findings. However, the size and heterogeneity of the complete sample, and our ability to stratify analyses based on key demographic factors while maintaining statistical power, are strengths. Future research will additionally address this limitation by longitudinally exploring NYCDOE FITNESSGRAM® flexibility and body composition data. Secondly, while FITNESSGRAM® measures collected in the school setting have been validated in other studies and populations [18, 45, 46], we do not have measures of the reliability or validity of the flexibility or BMI measures collected for this specific population and previous research in NYCDOE has demonstrated lower BMI data quality among high school students compared to K-8 graders [25, 34]. However, the extensive training and resources provided to the teachers (e.g. standardized stadiometer/scales) collecting this data likely enhances data quality among K-8 students. Finally, the FITNESSGRAM® includes the shoulder stretch as another possible test in its flexibility battery. However, this test is not used by NYCDOE, precluding our ability to examine its association with students’ weight status, thereby limiting our ability to contribute to the conversation about all flexibility tests.

## Conclusion

Data from a highly racially/ethnically and socioeconomically diverse sample of 4^th^-12^th^ grade students demonstrated a strong and consistent positive association between student weight status and trunk extension test performance: students with overweight or obesity perform better on the trunk extension test compared to students with normal weight. The opposite was observed for the sit-and-reach test, where across demographic groups, students with overweight and obesity had a lower odds of meeting age- and sex-specific sit-and-reach standards compared to normal weight students. Teachers, administrators, and researchers should be aware of the influence of BMI on student performance in these assessments.

These findings contribute important cross-sectional evidence to the ongoing debate on the use of criterion-referenced standard HFZ for flexibility to determine health-related physical fitness. These data suggest an advantage on the trunk extension test for students with overweight or obesity, calling into question the validity of this assessment as a measure of musculoskeletal flexibility. If this component of student health is to continue to be assessed, research on additional tests for trunk extensor strength and lumbar flexibility that do not favor heavier students, and that are feasible to conduct in a school setting, is warranted.

## Supporting information

S1 Table Sensitivity analysis—adjusted associations^A^ between student weight status^B^ and odds of being in the Healthy Fitness Zone^C^ for the FITNESSGRAM® Back-Saver Sit-and-Reach, defined as meeting standards for at least one side (left, right, or both), New York City 4^th^-12^th^ grade public school students, 2017–18, by student-level demographic characteristics. (DOCX)
