# Supplementary material for: The association between student body mass index and tests of flexibility assessed by the FITNESSGRAM®: New York City public school students, 2017–18
Source: PLoS One. 2021 Dec 31;16(12):e0262083. doi: 10.1371/journal.pone.0262083 (PMC8719681; doi:10.1371/journal.pone.0262083)
Supplement: S1 Table — (DOCX) [file pone.0262083.s001.docx]

**S1 Table: Sensitivity Analysis - Adjusted associations^A^ between student weight status^B^ and odds of being in the Healthy Fitness Zone^C^ for the FITNESSGRAM® Back-Saver Sit-and-Reach, defined as meeting standards for at least one side (left, right, or both),** **New York City 4^th^-12^th^ grade public school students, 2017-18, by student-level demographic characteristics**

|  | **Back-Saver Sit-and-Reach**  (n = 569,972 students) | | | | | |
| --- | --- | --- | --- | --- | --- | --- |
|  | **Underweight** | | **Overweight** | | **Obese** | |
|  | **# of students** | **OR** ± **SE**  **(95% CI)** | **# of students** | **OR** ± **SE**  **(95% CI)** | **# of students** | **OR** ± **SE**  **(95% CI)** |
| All students | 20,888 | 0.85 ± 0.01  (0.819, 0.873) | 103,763 | 0.84 ± 0.01  (0.822, 0.848) | 109,941 | 0.66 ± 0.01  (0.645, 0.666) |
| **Stratified models** |  |  |  |  |  |  |
| Females | 8,867 | 0.79 ± 0.02  (0.752, 0.829) | 52,437 | 0.83 ± 0.01  (0.809, 0.846) | 47,483 | 0.62 ± 0.01  (0.607, 0.636) |
| Males | 12,021 | 0.75 ± 0.02  (0.715, 0.782) | 51,326 | 0.86 ± 0.01  (0.836, 0.878) | 62,458 | 0.60 ± 0.01  (0.591, 0.618) |
| Elementary  (Grades 4-5) | 5,486 | 0.82 ± 0.03  (0.765, 0.875) | 25,611 | 0.79 ± 0.01  (0.761, 0.815) | 31,462 | 0.57 ± 0.01  (0.551, 0.588) |
| Middle School (Grades 6-8) | 6,449 | 0.77 ± 0.22  (0.732, 0.821) | 36,809 | 0.87 ± 0.01  (0.842, 0.890) | 39,597 | 0.66 ± 0.01  (0.647, 0.682) |
| High School (Grades 9-12) | 8,953 | 0.74 ± 0.02  (0.703, 0.777) | 41,343 | 0.85 ± 0.01  (0.824, 0.867) | 38,882 | 0.60 ± 0.01  (0.588, 0.620) |
| Asian/Pacific Islander | 6,859 | 0.76 ± 0.02  (0.722, 0.810) | 17,873 | 0.78 ± 0.01  (0.746, 0.807) | 12,346 | 0.59 ± 0.01  (0.567, 0.620) |
| Non-Hispanic black | 3,943 | 0.85 ± 0.03  (0.787, 0.913) | 23,287 | 0.85 ± 0.01  (0.819, 0.876) | 27,991 | 0.62 ± 0.01  (0.600, 0.639) |
| Hispanic | 5,544 | 0.82 ± 0.03  (0.769, 0.871) | 46,494 | 0.86 ± 0.01  (0.837, 0.879) | 55,569 | 0.62 ± 0.01  (0.602, 0.631) |
| Non-Hispanic white | 3,934 | 0.79 ± 0.03  (0.732, 0.849) | 14,412 | 0.82 ± 0.01  (0.790, 0.859) | 12,383 | 0.65 ± 0.01  (0.621, 0.679) |
| Not FRPM eligible | 6,226 | 0.76 ± 0.02  (0.720, 0.813) | 23,006 | 0.82 ± 0.01  (0.791, 0.847) | 20,928 | 0.63 ± 0.01  (0.613, 0.657) |
| FRPM eligible | 14,662 | 0.81 ± 0.02  (0.777, 0.839) | 80,757 | 0.83 ± 0.01  (0.819, 0.849) | 89,013 | 0.61 ± 0.01  (0.597, 0.619) |

^A^ Calculated from logistic mixed effects models with a random effect for school, adjusted for student-level: grade, race/ethnicity, free or reduced-price meal eligibility (a proxy for socioeconomic status), academic disability status, primary language spoken at home, and place of birth. The reference category for all models is students with normal weight.

^B^Weight status determined by student body mass index percentiles specific to age and sex calculated based on U.S. Center for Disease Control growth charts and weight status classifications. Being in the Healthy Fitness Zone for the Back-Saver Sit-and-Reach defined as meeting standards for at least one side (left, right, or both).

^C^ The FITNESSGRAM® uses Healthy Fitness Zones - criterion-referenced standards which represent minimum levels of fitness for age and sex that offer protection against the diseases that result from sedentary living – to evaluate fitness performance of students.

*Abbreviations: OR = Odds Ratio; SE = Standard Error; CI = Confidence Interval; FRPM = Free or reduced-price meal
